# Myocardial infarctions, subtypes and coronary atherosclerosis in SLE: a case–control study

**DOI:** 10.1136/lupus-2021-000515

**Published:** 2021-07-20

**Authors:** Isak Samuelsson, Ioannis Parodis, Iva Gunnarsson, Agneta Zickert, Claes Hofman-Bang, Håkan Wallén, Elisabet Svenungsson

**Affiliations:** 1 Unit of Rheumatology, Karolinska University Hospital, Stockholm, Sweden; 2 Department of Medicine, Solna, Karolinska Institutet, Stockholm, Sweden; 3 Unit of Cardiology, Danderyd Hospital, Stockholm, Sweden; 4 Department of Clinical Sciences, Danderyd Hospital, Karolinska Institutet, Stockholm, Sweden

**Keywords:** systemic lupus erythematosus, antiphospholipid syndrome, atherosclerosis, cardiovascular disease, inflammation

## Abstract

**Objective:**

Patients with SLE have increased risk of myocardial infarction (MI). Few studies have investigated the characteristics of SLE-related MIs. We compared characteristics of and risk factors for MI between SLE patients with MI (MI-SLE), MI patients without SLE (MI-non-SLE) and SLE patients without MI (non-MI-SLE) to understand underlying mechanisms.

**Methods:**

We identified patients with a first-time MI in the Karolinska SLE cohort. These patients were individually matched for age and gender with MI-non-SLE and non-MI-SLE controls in a ratio of 1:1:1. Retrospective medical file review was performed. Paired statistics were used as appropriate.

**Results:**

Thirty-four MI-SLE patients (88% females) with a median age of 61 years were included. These patients had increased number of coronary arteries involved (p=0.04), and ≥50% coronary atherosclerosis/occlusion was numerically more common compared with MI-non-SLE controls (88% vs 66%; p=0.07). The left anterior descending artery was most commonly involved (73% vs 59%; p=0.11) and decreased (<50%) left ventricular ejection fraction occurred with similar frequency in MI-SLE and MI-non-SLE patients (45% vs 36%; p=0.79). Cardiovascular disease (44%, 5.9%, 12%; p<0.001) and coronary artery disease (32%, 2.9%, 0%; p<0.001), excluding MI, preceded MI/inclusion more commonly in MI-SLE than in MI-non-SLE and non-MI-SLE patients, respectively. MI-SLE patients had lower plasma albumin levels than non-MI-SLE patients (35 (29–37) vs 40 (37–42) g/L; p=0.002).

**Conclusion:**

In the great majority of cases, MIs in SLE are associated with coronary atherosclerosis. Furthermore, MIs in SLE are commonly preceded by symptomatic vascular disease, calling for attentive surveillance of cardiovascular disease and its risk factors and early atheroprotective treatment.

**Key messages**

**What is already known about this subject?**

- Myocardial infarction (MI) is two to three times more common in patients with SLE as compared with age- and gender-matched community controls. Standardised incidence ratios for MI are even higher among younger patients with SLE.
- Subclinical atherosclerosis has been reported in many case–control studies, but a direct causal relationship between coronary artery disease (CAD) and MI in SLE has not yet been well documented.

**What does this study add?**

- In a majority (88%) of first-time MI-SLE cases, MIs were classified as MI with significant coronary artery disease.
- Symptomatic coronary artery disease preceded MI more commonly, and the number of involved coronary arteries was higher in patients with SLE compared with controls.

**How might this impact on clinical practice or future developments?**

- Early atheroprotective treatment and attentive surveillance for cardiovascular disease/CAD and associated risk factors are warranted in patients with SLE.

## Introduction

SLE is an autoimmune disease with heterogeneous presentation and a high female predominance (90%).^1^ Cardiovascular disease (CVD), usually defined as a combination of coronary, cerebrovascular and/or peripheral arterial disease, is a major cause of morbidity and premature mortality in SLE.^2–5^ Among the circulatory causes of death, ischaemic heart disease stands out as the major contributor.^3–5^ Overall, the incidence of myocardial infarction (MI) is estimated to be twofold to threefold elevated in SLE as compared with gender- and age-matched controls.^6 7^ Note that this figure is considerably higher in the younger age groups.^8 9^

Both traditional and lupus specific cardiovascular risk factors are believed to contribute to the high risk for MI. Renal involvement in patients with SLE has been associated with increased subclinical atherosclerosis^10^ and ischaemic heart disease.^11–13^ Fifteen to twenty per cent of SLE patients fulfil the criteria for antiphospholipid syndrome (APS),^14^ defined by venous, arterial or small vessel thrombosis and/or obstetric complications together with persistent positivity for antiphospholipid antibodies (aPL).^15^ Venous thromboembolism (VTE) and aPL have been associated with CVD in SLE^9 16–20^ similarly to high daily intake of glucocorticoids^17^ and low plasma albumin levels.^16^ Note that these four risk factors remain to be verified specifically for MI in SLE.

Though several risk factors have been suggested, the exact mechanisms behind the high MI incidence in SLE remain essentially unknown. Coronary atherosclerosis is often assumed to be the cause of MIs, since high rates of subclinical atherosclerosis have been reported in patients with SLE.^21–26^ Yet, few studies have directly investigated the extent of coronary atherosclerosis at the time of the MI events.^27^

In this case control study, we compare MI subtypes between patients with or without SLE, in order to determine if coronary atherosclerosis contributes to SLE-associated MIs to the same extent as to MIs in community controls. In addition, we identify risk factors using MI, rather than the broader CVD, as outcome.

## Materials and methods

### Patients and controls

Patients with SLE were recruited between January 1995 and December 2017 through use of the Karolinska SLE cohort,^28^ which includes patients from the Karolinska University Hospital and Danderyd Hospital in Stockholm, Sweden. Patients aged 20–85 years with SLE according to the revised 1982 American College of Rheumatology SLE classification criteria^29^ and a first-time MI according to the Third Universal Definition of MI^30^ were included.

We used the national Swedish Web-system for Enhancement and Development of Evidence-based care in Heart disease Evaluated According to Recommended Therapies (SWEDEHEART) registry^31^ and the Swedish Myocardial Infarction Registry (Riks-HIA), which is a subregistry of SWEDEHEART, to identify patients with a first-time MI but not SLE (MI-non-SLE) as comparators. The SWEDEHEART registry covers approximately 85% of Swedish patients with MI. Riks-HIA contains clinical data related to hospitalisations due to acute coronary syndrome.^31^

MI-SLE patients were individually matched for (1) gender, (2) age and (3) date of MI event to MI-non-SLE patients in a ratio of 1:1. Using similar matching, we identified a second set of controls with SLE but no history of MI (non-MI-SLE) from the Karolinska SLE cohort.^28^ In this manner, triplets were created, each including one MI-SLE patient, one MI-non-SLE patient and one non-MI-SLE patient in a ratio of 1:1:1. Patients and controls were all living in Stockholm county. We did not match for different regions within Stockholm.

### Definition of events and risk factors

Data were collected through retrospective medical file review in all patients with MI type 1–3 according to Thygesen *et al*.^30^ See table 1 for detailed descriptions of the chosen variables. Supportive medical files including laboratory tests, renal biopsies, imaging, electrophysiological examinations or appropriate treatment were required in order to classify patients as being diseased. Data regarding serum/plasma creatinine and albumin levels were collected from medical files when available within 3 months before study entry. Estimated glomerular filtration rate (eGFR) was calculated using the re-expressed four-variable Modification of Diet in Renal Disease (MDRD) study equation according to Levey *et al.*^32^ Diabetes and SLE renal involvement was defined according to guidelines from the American Diabetes Association^33^ or Tan *et al*,^29^ respectively.

**Table 1:** Detailed description of variables

	Description and comments	Diagnostics	Treatment
ST-elevation MI (STEMI)	As by medical file review. In patients with pacemaker and/or ECG findings of ventricular rhythm, preexcitation or left bundle branch block, data were considered missing, as data from previous ECGs were not available in most cases.	ECG.	N/A
Non-STEMI	As above.	ECG.	N/A
MI with coronary artery disease (MI-CAD)	≥50% coronary stenosis/occlusion at the event of MI according to Agewall *et al.*^45^	Coronary angiography.	N/A
0-vessel disease (0-VD)	<50% coronary stenosis and/or occlusion at the event of MI according to Agewall *et al*^45^ Also referred to as MI with non-obstructive coronary arteries (MINOCA).	Coronary angiography.	N/A
Number of involved coronary arteries	Numbered 0-VD to 3-VD depending on how many out of LAD, RCA and/or Cx that had ≥50% coronary stenosis/occlusion. LMCA was classified as 2-VD, as it most commonly branches to LAD and Cx.	Coronary angiography.	N/A
Involvement of specific coronary arteries	≥50% coronary stenosis/occlusion in LMCA, LAD, RCA, Cx and/or any of their respective branches.	Coronary angiography.	N/A
Left ventricular ejection fraction	As by medical file review.	Echocardiography.	N/A
Coronary artery disease (CAD)	Stable CAD or unstable angina were not separated. MI was not considered.	Coronary angiography.	Antiplatelet agents or nitroglycerin prescription, CABG or PCI.
Ischaemic stroke	Haemorrhagic stroke was not considered.	CT or MRI.	Antiplatelet agents or thrombolytic therapy.
Peripheral arterial diseases (PADs)	All types of PAD were considered.	CT, DUS, invasive angiography and toe pressure,	Antiplatelet agents, PTA or surgery including bypass grafting.
Cardiovascular disease (CVD)	Stable CAD, unstable angina, ischaemic stroke or PADs.	As stated above.	As above.
Venous thromboembolism (VTE)	All types of venous thrombosis including pulmonary embolism were considered as VTE.	CT, DUS, MRI or phlebography depending on VTE location.	LMWH, OAC prescription, unfractioned heparin.
Antiphospholipid syndrome	Clinical manifestations of thrombosis, that is, stroke and/or VTE, and/or obstetric complications according to Miyakis *et al*,^15^ with the modification that we only required aPL-positivity on one occasion, as many patients lacked repeated measurements.	See ischaemic stroke and/or VTE for specifics on these variables.	See ischaemic stroke and/or VTE for specifics on these variables.
Diabetes	Diabetes types 1 and 2 were not separated.	Fasting plasma glucose ≥126 mg/dL (7.0 mmol/L), 2-hour plasma glucose ≥200 mg/dL (11.1 mmol/L) during oral glucose tolerance testing, HbA1c ≥6.5% (48 mmol/mol) and/or a random plasma glucose ≥200 mg/dL (11.1 mmol/L) in patients with classic symptoms of hyperglycaemic according to American Diabetes Association in 2018.^33^	Insulin or other antidiabetic agents.
Renal involvement	As by medical file review.	Renal biopsy, proteinuria and/or cellular cast according to Tan *et al*.^29^	Cyclophosphamide, mycophenolate mofetil or azathioprine treatment. Dialysis or kidney transplantation.

CABG, coronary bypass grafting; DUS, duplex ultrasound; LAD, left anterior descending artery; LMCA, left main coronary artery; LMWH, low molecular weight heparin; N/A, not applicable; OAC, oral anticoagulants; PCI, percutaneous coronary intervention; PTA, percutaneous transluminal angioplasty; RCA, right coronary artery; 0-VD to 3-VD, 0 to 3-vessel disease.

### Statistical analysis

Median and IQR were used to describe continuous variables. For comparison between matched pairs, the McNemar’s and the Wilcoxon signed-rank tests were used for binominal or ordinal and continuous data, respectively. When analysing triplets, the Cochran’s Q test and the Friedman’s test were used for binominal or ordinal and continuous data, respectively. Statistical analysis was performed using the IBM SPSS Statistics software, V.25 (IBM Corporation). P values <0.05 were considered statistically significant.

### Ethics

Written informed consent was obtained from all patients in the Karolinska SLE cohort. In order to enable nationwide coverage, participants in the population-based Riks-HIA registry are informed about their inclusion and their possibility to opt-out. They are also informed that information in the registry may be used in studies approved by ethical committees.

### Patient and public involvement

Neither members of the public nor patients with SLE were involved in the design or conduction of this study.

## Results

### Baseline characteristics

We identified 34 SLE patients with type 1–3 MI according to Thygesen *et al.*^30^ These 34 patients were matched to controls and further studied. Median age at study entry and female prevalence were equal between the matched MI-SLE, MI-non-SLE and non-MI-SLE patients, that is, 61 years or 88%, respectively. Median age at SLE onset was 36 (22–47) years and 42 (27–53) years in MI-SLE or non-MI-SLE patients, respectively (p=0.08). Baseline characteristics are presented in table 2.

**Table 2:** Baseline characteristics and risk factors for myocardial infarction (MI) in patients with and without SLE (for detailed descriptions of variables see table 1)

	MI-SLE	MI-non-SLE	Non-MI-SLE	P value*	Post hoc analysis*
	N=34	N=34	N=34		
Women, n (%)	30 (88)	30 (88)	30 (88)		
Age, median (IQR) years	61 (49–68)	61 (49–69)	61 (49–68)		
Age at SLE onset, median (IQR) years	36 (22–47)		42 (27–53)	0.08	
CVD and VTE combined, n (%)	4 (12)	0 (0)	1 (2.9)	0.07	
Venous thromboembolism (VTE), n (%)	9 (26)	0 (0)	5 (15)	0.004	P=0.29††
Cardiovascular disease (CVD, MI excluded), n (%)	15 (44)	2 (5.9)	4 (12)	<0.001	P=0.007††
Coronary artery disease (MI excluded), n (%)	11 (32)	1 (2.9)	0 (0)	<0.001	P=0.002‡‡
Ischaemic stroke, n (%)	6 (18)	1 (2.9)	2 (5.9)	0.10	
Peripheral arterial diseases, n (%)	6 (18)	2 (5.9)	2 (5.9)	0.17	
Diabetes, n (%)	4 (12)	3 (8.8)	0 (0)	0.16	
Antiphospholipid syndrome, n (%)	7 (21)	0 (0)	4 (12)	0.04	P=0.55††
Smoking status, n (%)					
- Never smoker	6 (18)	15 (44)	12 (35)		
- Previous smoker	18 (53)	8 (24)	18 (53)	0.18	P=0.34§§
- Current smoker	8 (24)	11 (32)	4 (12)		§§
- Missing	2 (5.9)	0 (0.0)	0 (0.0)		
Prednisolone equivalents, median (IQR) mg/24 hours	5.0 (0.0–7.5)	0.0 (0.0–0.0)	2.5 (0.0–7.5)	<0.001†	P=0.97††
Hydroxychloroquine at MI/inclusion, n (%)	7 (23)		18 (53)	0.06‡	
Low-dose acetylsalicylic acid at MI/inclusion, n (%)	11 (36)		3 (10)	0.04§	
Creatinine, median (IQR) mmol/L	81 (68–100)	70 (59–79)	72 (58–88)	0.09¶	
Creatinine-based eGFR, median (IQR) mL/min/1.73 m^2^	67 (48–77)	79 (67–92)	72 (60–106)	0.08¶	
Current/previous renal involvement, n (%)	15 (44)		11 (32)	0.42	
Albumin levels in blood, median (IQR) g/L	35 (29–37)		40 (37–42)	0.002**	

*P values are derived from comparing matched triplets using the Cochran’s Q and the Friedman’s test for binominal or ordinal and continuous data, respectively. For comparison between matched pairs, the McNemar’s and the Wilcoxon signed-rank tests were used for binominal and continuous data, respectively.

†Data were available in 34/34 MI-SLE, 33/34 MI-non-SLE and 34/34 non-MI-SLE patients.

‡Data were available in 31/34 MI-SLE and 34/34 non-MI-SLE patients.

§Data were available in 31/34 and 31/34 non-MI-SLE patients.

¶Data were available in 32/34 MI-SLE, 28/34 MI-non-SLE and 34/34 non-MI-SLE patients.

**Data were available in 16/34 MI-SLE and 34/34 non-MI-SLE patients.

††A post hoc comparison between MI-SLE and non-MI-SLE.

‡‡A post hoc comparison between MI-SLE and MI-non-SLE.

§§A post hoc comparison between the frequency of currently smoking MI-SLE patients and the frequency of currently smoking non-MI-SLE patients.

CAD, coronary artery disease (MI excluded); CVD, cardiovascular disease; eGFR, estimated glomerular filtration rate; PADs, peripheral arterial diseases; VTE, venous thromboembolism.

### Traditional risk factors

Neither smoking status (table 2; p=0.18) nor diabetes (12%, 8.8%, 0%; p=0.16) differed significantly between MI-SLE, MI-non-SLE or non-MI-SLE patients, respectively. Traditional risk factors are presented in table 2.

### Lupus specific risk factors

Low plasma albumin levels distinguished MI-SLE patients from non-MI-SLE patients (35 vs 40 g/L; p=0.002). Both plasma creatinine (81, 70 and 72 mmol/L; p=0.09) and creatinine-based eGFR (67, 79 and 72 mL/min/1.73 m^2^; p=0.08) indicated a non-significant trend towards impaired renal function in MI-SLE as compared with MI-non-SLE and non-MI-SLE patients. However, the prevalence of current/previous renal involvement did not differ significantly between MI-SLE patients and non-MI-SLE patients (44% vs 32%; p=0.42). Previous VTE and APS did not distinguish MI-SLE patients from controls, as neither VTE (26% vs 15%; p=0.29) nor APS (21% vs 12%; p=0.55) differed significantly between MI-SLE and non-MI-SLE patients. Lupus specific risk factors are presented in table 2.

### Medications

Twenty-three per cent of MI-SLE patients and 53% of non-MI-SLE controls were prescribed hydroxychloroquine (HCQ) at MI or study entry, respectively (p=0.06). Thirty-six per cent of MI-SLE patients and 10% of non-MI-SLE controls were prescribed low-dose acetylsalicylic acid (ldASA) at MI or study entry, respectively (p=0.04). Median daily intake of prednisolone equivalents at hospital admission did not distinguish MI-SLE patients from non-MI-SLE controls (5 vs 2.5 mg; p=0.97). Medications are presented in table 2.

### History of cardiovascular diseases

Symptomatic CVD, defined as either CAD (MI excluded), ischaemic stroke or peripheral arterial diseases (PADs), preceded the MI event/inclusion more commonly in MI-SLE patients compared with MI-non-SLE and non-MI-SLE patients (44%, 5.9% or 12%; p<0.001). When stratifying for subtypes of CVD, only CAD was significantly more common in MI-SLE patients compared with MI-non-SLE and non-MI-SLE patients: CAD (32%, 2.9% and 0%; p<0.001), ischaemic stroke (18%, 2.9% and 5.9%; p=0.10) and PADs (18%, 5.9% and 5.9%; p=0.17), respectively.

### MI characteristics

In types 1–3 MIs, frequency of ST-elevation MI (STEMI) was similar between MI-SLE patients and MI-non-SLE patients (28% vs 34%; p=1.0). MI with coronary artery disease (MI-CAD), that is, ≥50% coronary stenosis/occlusion at the event of MI, was present in 88% and 66% of MI-SLE patients or MI-non-SLE patients, respectively (p=0.07). MI-SLE patients had a higher number of coronary arteries involved at MI compared with controls (table 3; p=0.04). The left anterior descending artery (LAD) was most frequently involved in both MI-SLE and MI-non-SLE patients (73% vs 59%; p=0.11) followed by the right coronary artery (27% vs 31%; p=0.75) and the circumflex artery (Cx) (23% vs 21%; p=1.0). The frequency of impaired left ventricular ejection fraction (LVEF), that is, <50%, after MI did not differ significantly between MI-SLE and MI-non-SLE patients (45% vs 36%; p=0.79). MI characteristics are presented in table 3.

**Table 3:** Findings according to ECG, coronary angiography and echocardiography in MI patients with or without SLE

	MI-SLE, n (%)	N_total_	MI-non-SLE, n (%)	N_total_	P value
NSTEMI	23 (72)	32	21 (66)	32	1.0*
STEMI	9 (28)	32	11 (34)	32	
MINOCA (0-VD)	3 (12)	26	10 (35)	29	0.07*
MI-CAD (≥1 VD)	23 (88)	26	19 (66)	29	
0-VD	3 (12)	26	10 (35)	29	0.04†
1-VD	13 (50)	26	9 (31)	29	
≥2-VD	10 (39)	26	10 (35)	29	
LMCA involvement	3 (12)	26	0 (0)	29	0.50*
LAD involvement	19 (73)	26	17 (59)	29	0.11*
RCA involvement	7 (27)	26	9 (31)	29	0.75*
Cx involvement	6 (23)	26	6 (21)	29	1.0*
LVEF <50%	13 (45)	29	12 (36)	33	0.79*
LVEF ≥50%	16 (55)	29	21 (64)	33	

*The McNemar test was used.

†The Wilcoxon signed-rank test was used.

Cx, circumflex artery; LAD, left anterior descending artery; LMCA, left main coronary artery; LVEF, left ventricular ejection fraction; MI-CAD, MI with coronary artery disease; MINOCA, MI with non-obstructive coronary arteries; MI-non-SLE, MI patients without SLE; MI-SLE, MI patients with SLE; NSTEMI, non-ST-elevation MI; RCA, right coronary artery; STEMI, ST-elevation MI; 0-VD, 0-vessel disease; 1-VD, 1-vessel disease; 2-VD, 2-vessel disease.

## Discussion

In this study, 88% of MIs in patients with SLE were classified as MI-CADs. The number of involved coronary arteries at MI were higher, and symptomatic CAD preceding MI was more common in MI-SLE patients as compared with age-matched and gender-matched MI controls. These findings suggest that accelerated coronary atherosclerosis is a major cause of the increased risk of MI, previously documented in patients with SLE.^6–9^

Studies of atherosclerosis using coronary angiography in patients with SLE are to date few. In a registry-based study by Tornvall *et al*,^27^ the frequency of MI-CAD was similar in patients with SLE and controls, but patients with SLE were 7 years younger at their first MI. Kaul *et al*^21^ used a different approach and compared 86 patients with SLE with 258 sex-matched and year of catheterisation-matched controls, who had been subject to coronary angiography on clinical indications. Patients with SLE demonstrated similar rates of CAD as controls, despite the fact that the median age was 21 years younger in patients with SLE as compared with the controls (49 vs 70 years). CAD was associated with SLE after adjustment for traditional cardiovascular risk factors including age. Sella *et al*^34^ performed coronary angiography in 21 patients with SLE and reported that atherosclerotic plaques were, besides traditional cardiovascular risk factors, associated with higher SLE disease activity.^34^ Taken together, these results support a pivotal role for coronary atherosclerosis as a risk factor for MI in patients with SLE.

In conformity with previous reports,^21 34^ LAD was the most commonly involved artery in our study. LAD involvement may lead to complications related to left ventricular dysfunction. The prevalence of impaired LVEF did not differ significantly between MI-SLE and MI-non-SLE patients in this study, but it is known that the risk of heart failure (HF) is increased in SLE.^35 36^ Note that subtypes of HF have not been separated in previous studies. Therefore, further studies are warranted, in order to better understand the relative contribution of MI-CAD and LAD-involvement, as HF with reduced EF, rather than HF with preserved EF, is typically associated with MI-CAD.

Besides CAD, symptomatic CVD preceded MI more often in MI-SLE patients than in controls. Also, ldASA before MI/inclusion was more commonly prescribed to MI-SLE patients compared with controls. This finding is expected, since ldASA is used as secondary prevention for both CVD and CAD. We were not able to specifically investigate whether atherosclerosis was generalised or not. However, previous studies have demonstrated evidence of atherosclerotic plaques also in non-cardiac vascular locations in patients with SLE.^22 25 26^ These results also indicate accelerated atherosclerosis in SLE and call for attentive surveillance and early initiation of antiatherosclerotic prophylactic treatment in patients with SLE. To date, treatment studies in SLE are few and underpowered, but a meta-analysis of eight trials with statins demonstrated lower lipid and lower CRP levels in patients undergoing statin treatment.^37^

Besides statins, HCQ use may reduce the risk of MI in patients with SLE. Even though the difference in HCQ use between MI-SLE patients and non-MI-SLE was only trendwise significant in the present study, other authors have reported an atheroprotective effect related to HCQ use in patients with SLE.^38 39^ In a meta-analysis by Liu *et al*,^38^ HCQ use was associated with a 36% (95% CI 0.51 to 0.81) lower relative risk of CVD in patients with SLE. Besides demonstrating lower HRs for major adverse cardiovascular events, Haugaard *et al*^39^ found a 49% (95% CI 0.27 to 0.97) lower HR for MI in SLE patients with HCQ compared with controls. Note that MI was a secondary outcome in the study by Haugaard *et al*.^39^ HCQ has a known lipid-lowering effect^40^ and ameliorate inflammation in SLE^41^ partly through decreased interleukin (IL)-1β and IL-6 production.^42^ These cytokines have been attributed a casual role in the process of atherosclerosis.^43 44^ Taken together, the result from the present study is in line with larger prospective and mechanistic studies, which suggest an atheroprotective effect from HCQ use in patients with SLE.

MI with non-obstructive coronary arteries (MINOCA), defined as <50% coronary stenosis/occlusion,^45^ was trend wise more prevalent in our MI-non-SLE controls as compared with MI-SLE patients (35% vs 12%; p=0.07). The prevalence of MINOCA in MI-SLE patients was similar in our study and the study by Tornvall *et al*,^27^ that is, 12% versus 11%, respectively. In a meta-analysis by Pasupathy *et al*,^46^ MINOCA frequency in the general population was lower than in our MI-non-SLE controls: 6% versus 35%, respectively. The fact that our MI-non-SLE controls were individually matched to the MI-SLE patients yielded an enrichment of young females among MI-non-SLE controls. Both younger age and female gender are known to be associated with an increased risk of MINOCA.^46^ This likely explains the higher MINOCA frequency observed in our MI-non-SLE controls.^46^ Because our data were collected retrospectively from medical files of standard MI care, our classifications are based on one interventionist’s opinion. By contrast, the diagnosis of MINOCA usually requires the opinion of two independent interventionists when used for scientific purposes. This may further contribute to the discrepancy in MINOCA prevalence reported in our study and the study by Pasupathy *et al*.^46^

Although VTE and APS were not significantly associated with MI in the present study, previous studies have demonstrated that aPL predict CVD defined as a composite outcome.^9 16–20^ It is worth noting that aPL has been more strongly linked to stroke and VTE than to MI.^47^ This is one possible explanation for the discrepancy between our and the previous studies, since the present study is focused on MI.

Renal involvement has previously been identified as a risk factor for MI in patients with SLE.^11–13^ We have previously reported that excess atherosclerosis, as indicated by carotid plaque occurrence, was restricted to patients with lupus nephritis rather than patients without lupus nephritis.^10^ In the current study, we observed a trend towards lower eGFR in MI-SLE patients. We could not replicate previously reported associations between renal involvement and MI in patients with SLE,^11–13 27^ possibly due to lack of power. Similarly, smoking status and diabetes were not associated with MI, even though these variables have been previously linked to CVD in SLE.^16 18–20^ Age at SLE onset differed numerically, but not statistically, when MI-SLE patients were compared with non-MI-SLE patients, also possibly due to a small sample size.

When possible to retrieve, we noticed significantly lower plasma albumin levels collected within 3 months of study entry in MI-SLE patients compared with matched non-MI-SLE controls. We and others have previously proposed that albumin is an inverse surrogate marker of disease activity in SLE, in particular in patients with renal disease, who lose albumin in the urine.^28 48^ An increased cumulative inflammatory burden, as indicated by lower albumin levels in MI-SLE, may increase the risk of MI through several mechanisms. Accumulated flares or a steady-state inflammatory milieu may over time, together with certain treatments, for example, glucocorticoids, increase the risk of MI.^17^ These factors, together with renal involvement may collectively contribute to accelerate atherosclerosis and/or make atherosclerotic plaques more prone to rupture, as discussed in two systematic reviews.^49 50^ It is worth noting that corticosteroid dose at hospital admission was not associated with MI in the present study. However, in contrast to Magder *et al*,^17^ we did not have data on accumulated exposure to glucocorticoids, which normally increases with disease duration.

This study has strengths. First, we used well-defined MI as our primary outcome rather than a mixture of vascular outcomes. Second, patients with SLE were recruited from a large consecutively collected cohort of well-characterised patients. We also acknowledge limitations, such as the retrospective design, the limited power for statistical analysis and missing data in some cases, for example, data on coronary angiography findings and plasma albumin levels. Furthermore, it is possible that comorbidities were more commonly identified before the event in MI-SLE patients because of closer surveillance of these patients as compared with the general population comparators.

## Conclusions

The present study demonstrates that MIs in SLE are in most cases associated with coronary atherosclerosis. MIs in SLE were also often preceded by symptomatic vascular disease, calling for more attentive surveillance of CVD and its risk factors as well as for early atheroprotective interventions. Further larger and preferable prospective studies are needed in order to understand the specific causes of accelerated coronary atherosclerosis in SLE.

## Data Availability

Data are available on reasonable request. Deidentfied participant data are available on reasonable request.
